# Domenique Jean Larrey, 1766-1842

**Published:** 1956-10

**Authors:** A. E. S. Roberts


### DOMENIQUE JEAN LARREY, 1766–1842

BY

A. E. S. ROBERTS

There is in the Medical Library a small seventeen-page treatise entitled *Mémoire sur l'ophthalmie regnante en Egypte, Par le Citoyen Larrey, Chirurgien en Chef de L'Armée d'Orient, 1802*. It was written and published during the short period the French were in occupation of Egypt. This historic pamphlet on ophthalmia describes a disease which is mentioned in the Ebers Papyrus but which remained unnoticed until Larrey saw it some 3,000-odd years later. He seems to have been the first to point out the contagious nature of this disease, and it is this observation which establishes the historical importance of this little work. But what makes this particular copy more interesting is that it bears the signature of Larrey and that in addition he has written on the blank last page "Les oiseaux et les animaux domestiques vient sujects à cette maladie."

Domenique Jean Larrey (1766–1842) stands alone as the greatest military surgeon in history. He was born during the most degraded, degenerated, corrupt and miserable period of modern French history in a small village in the Pyrenees, and after studying at Toulouse and Paris he eventually joined the Army of the Rhine at Strasbourg as surgeon. He later became chief surgeon to the Army in Italy and Napoleon gave him *carte blanche* in the organization of the medical division. He was with Napoleon at all his battles, and on his return to Paris from Egypt in 1801 honours were showered upon him. During the pauses between campaigns he gave courses of lectures and demonstrations and opened military medical schools in France, Spain and Germany. It is probably due to Larrey's constant work, both in teaching and organizing, that the French Army surgeons were the best in the world at this time. After the fall of Napoleon Larrey was stripped of his honours, wealth and position and barely escaped with his life. He was asked by Alexander to go to Russia to organize the medical department of his army, and Don Pedro did his utmost to induce him to emigrate to Brazil. However, in 1818 honours came again to him and he was restored to his old position in the Army, and when in 1826 he visited England, the country's greatest men vied with one another to do him homage. He was invited to Belgium by the king of that country to advise on army hospitals, and in 1834 he toured southern France and Italy. He died of pneumonia on his way home from Algiers where he had been sent to investigate some trouble which had broken out among the conscripts.

Had he done nothing more than devise the "flying ambulance" his would have been one of the illustrious names in military surgery. The old cumbersome vehicles, which required forty horses to move one of them, and owing to their size were required to remain three miles behind the firing line, were replaced by light vehicles which could go within one hundred feet of the firing line even during action and which could immediately transport the wounded to the hospitals. At Austerlitz, the bloodiest of Napoleon's conflicts, all the wounded, friend and foe alike, were operated upon, their wounds dressed, and in the hospitals in less than twenty-four hours. Larrey's treatment of the wounded was radical and diametrically opposed to precedent, and while primary amputation did not originate with him, he was the first European military surgeon to advocate the method. Amputation immediately after the injury originated with English naval surgeons in the seventeenth century, and is first plainly described by Wiseman in 1676. Immediate amputation became the general practice in the British Navy during the eighteenth century. But this was not the case with military surgeons, especially in France. Except for Le Dran, all important French surgeons recommended delay. Fully 75 per cent. of the immediate amputations performed by Larrey and his aides recovered, an astonishing figure when it is recalled that this included all causes of death. His treatment of burns by forced feeding and stimulation excited the ridicule and opprobrium of contemporary surgeons who advocated bleeding and a greatly reduced diet. But Larrey's burn patients continued to improve. Cleanliness, simplicity and rest were his trinity of treatment. The most important of his achievements may be summed up as follows:

1. Use of salt solution in simple wounds and of Labarraque's solution in cases of infection.
2. Avoidance of all greases, ointments or elaborate dressings.
3. Avoidance of all meddlesome surgery.
4. Insistence upon rest for all wounds by not too frequent dressings.
5. Resection of ribs for empyema.
6. Supportive treatment for burns.
7. Immediate amputations.
8. Resection in selected cases.
9. Removal of sick and wounded to a base hospital as soon as possible.
10. "*Ambulances volantes*".

### REFERENCES

Gubbins, L. (1914). *J. Roy. Army Med. Corps*, **22**, 186.
Da Costa, J. C. (1906). *Johns Hopk. Hospl. Bull.*, **17**, 195.
Da Costa, J. C. (1915). *Brit. Med. J.* **1**, 265, 1056.
Coues, W. P. (1920). *Boston Med. and Surg. J.*, **182**, 146.
Coues, W. P. (1926). *Ibid.*, **194**, 21.
Marcy, H. O. (1920). *Ibid.*, **182**, 310.
King, H. D. (1917). *J.A.M.A.*, **69**, 1106.
Spencer, W. G. (1919). *Lancet*, **1**, 867, 920, 962.
Spencer, W. G. (1917). *J.A.M.A.* **69**, 1084.

